# An experimental *in-vivo *canine model for adult shunt infection

**DOI:** 10.1186/1743-8454-5-17

**Published:** 2008-10-24

**Authors:** Roger Bayston, Christine Brant, Stephen M Dombrowski, Geraldine Hall, Marion Tuohy, Gary Procop, Mark G Luciano

**Affiliations:** 1 BRIG, Division of Orthopaedic and Accident Surgery, C Floor West Block, Queen's Medical Centre, University of Nottingham, Nottingham, NG7 2UH, UK; 2 Department of Neurosurgery – S80, the Cleveland Clinic Foundation, 9500 Euclid Avenue, Cleveland, OH, 44195, USA; 3 Department of Clinical Pathology – L40 the Cleveland Clinic Foundation, 9500 Euclid Avenue, Cleveland, OH, 44195, USA

## Abstract

**Background:**

Detailed human studies of the mechanisms and development of shunt infection in real time are not possible, and we have developed a canine hydrocephalus model to overcome this. The intention of this pilot study was to show that the canine hydrocephalus model could be shunted using conventional "human" shunts, and that a shunt infection could be established so that further studies could then be planned.

**Methods:**

Hydrocephalus was induced in seven dogs (*Canis familiaris*) by fourth ventricle obstruction. Four weeks later they were shunted using a Hakim Precision valve. Four of the dogs received shunts whose ventricular catheter had been inoculated with *Staphylococcus epidermidis*, and three were uninoculated controls. Four weeks after shunting the dogs were sacrificed and necropsy was performed. Removed shunts and tissue samples were examined microbiologically and isolates were subjected to detailed identification and genomic comparison.

**Results:**

All the dogs remained well after shunting. Examination of removed shunt components revealed *S. epidermidis *in the brain and throughout the shunt system in the four inoculated animals, but in two of these *Staphylococcus intermedius *was also found. *S. intermedius *was also isolated from all three "negative" controls. There were slight differences between *S. intermedius *strains suggesting endogenous infection rather than cross- infection from a point source.

**Conclusion:**

Shunt infection was established in the canine model, and had the experiment been extended beyond four weeks the typical microbiological, pathological and clinical features might have appeared. The occurrence of unplanned shunt infections in control animals due to canine normal skin flora reflects human clinical experience and underlines the usual source of bacteria causing shunt infection.

## Background

The introduction of cerebrospinal fluid (CSF) shunts revolutionized the treatment of hydrocephalus and greatly decreased mortality rate. However, over subsequent decades, difficulties in shunt maintenance and problems resulting from infection or occlusion have persisted [1]. Today, shunt failure rates range from 25% to 40% in the first few months after surgery [2,3]. After this critical period, the risk remains at 4% – 5%. As a result, some patients may have numerous shunt revisions during their lifetime, representing a major medical risk. Infections have been a leading cause of shunt failure with infection rates ranging from 1% to 30% [4-8]. Overall, the international reported incidence is around 10% [9]. Gram positive bacteria are most commonly implicated, and approximately 80% are caused by staphylococci, most of these being *Staphylococcus epidermidis *[9,10]. These organisms originate on the patient's skin and mucous membranes and investigational and clinical evidence indicates that the inoculation of the shunt system occurs at the time of operation [11]. The severity of a shunt infection may vary from a sub-clinical colonization of the shunt inner lumen surface to fulminating ventriculitis, meningitis, or encephalitis which can be neurologically catastrophic, although the picture with *S. epidermidis *infections is typically low-grade. A shunt-related infection of the central nervous system (CNS) is of particular concern because of the threat to cerebral functions. Treatment may be prolonged and relapses may occur, complicating control of intracranial pressure and giving rise to life-threatening risks such as cognitive and neurological deficits [3,6,12,13].

Although many studies have investigated the rate, aetiology and epidemiology of shunt infections, preventive measures are difficult to study, and their pathophysiology is largely unknown due to the difficulties inherent in human studies. Animal models of infection in other implantable devices have not hitherto been suitable for these purposes. We have developed a canine model of chronic obstructive hydrocephalus [14] which produces a gradual, well – tolerated increase in ventricular size, resulting in transient and subtle clinical effects. Unlike previous models, there is no focal compression or general inflammation which may confound analysis [15,16]. Because of the size of the animals this model allows actual shunting procedures to be carried out using shunts designed for human use, and clinically relevant measurements to be made for the purposes of diagnosis, treatment and research. This paper, therefore, describes and characterises an animal model able to address these issues, and reports its use in a pilot study of chronic low-grade shunt infection.

## Methods

### Animals and induction of hydrocephalus

Seven young adult (8–9 months) male purpose – bred mongrel dogs (*Canis familiaris*) weighing 25–30 kg were used for this study. Animals were obtained from licensed suppliers and quarantined for a minimum of seven days before entering the study. They were maintained in the Cleveland Clinic's fully accredited Animal Care Facility and their maintenance and all experimental procedures were conducted in accordance with the National Institutes of Health (NIH) and The Cleveland Clinic Foundation Animal Research Committee (ARC) guidelines for the use and care of laboratory animals. Each experiment began with the induction of obstructive hydrocephalus (time zero), the details of which have been previously described [14,17]. General endotracheal anaesthesia by 1% isoflurane gas inhalation was used for all surgeries. Briefly, a midline suboccipital craniotomy was performed enabling the skin and muscles to be retracted and the dura opened along the midline. Animals were given gentamicin (40 mg i.v.) and cefazolin (1 g i.v.) prophylactically. The cerebellar vermis and tonsil were exposed and retracted upward, to allow a flexible silicone catheter (~1.5 mm diameter) connected to a 1 mL syringe to be inserted through the foramen of Magendie into the fourth ventricle. Cyanoacrylic gel (0.4 – 0.6 mL) was injected into the fourth ventricle to obstruct the normal flow of CSF. The catheter was left in position after being cut at the level of the foramen of Magendie, and the dura was closed with 5-0 Ethibond. After muscle, fascia, and skin were closed in layers, each animal was admitted to intensive care for recovery (24–72 hours). All the animals received Tylenol for pain. Severe pain was controlled with torbutrol (0.4 mg/kg, i.m. b.i.d. for 5 days). In the event that an animal did not eat and was losing weight, subcutaneous dextrose feeding was administered. In addition, if any animal presented with symptoms of acute hydrocephalus, medical treatment for increased intracranial pressure (ICP) including mannitol (0.25–1.0 g/kg), diamox (125 mg), and decadron (4–6 mg) was administered.

### Shunt insertion and inoculation

Approximately four weeks after induction, the hydrocephalic animals underwent a standard shunt implantation surgery. The preoperative method of sedation and positioning with the stereotaxic head frame was the same as described previously [14,17]. The animals did not receive any antibiotic prophylaxis before or after surgery for implantation of the shunt system and inoculation. Ventriculomegaly was confirmed by MRI [14]. Images were obtained at baseline and at time of sacrifice, and were compared using planimetric measures (i.e. Evans ratio). Overall, all animals induced with chronic hydrocephalus had an Evans ratio >0.3. However, the degree or severity of hydrocephalus was not relevant for this study, and all animals were surgically implanted with a ventriculoperitoneal (VP) shunt regardless of the degree of ventriculomegaly. A frontal burr hole craniotomy was performed and a standard silicone shunt with a one-way pressure valve was used for the VP shunt procedure (HAKIM Precision Valve Standard without prechamber, opening pressure: 10 ± 10 mm H_2_O, Codman and Shurtleff Inc, Raynham, MA, USA). Prior to placement, the ventricular catheter in four of the animals was inoculated with *S. epidermidis sensu stricto*, F22, a clinical isolate from a proven VP shunt infection. A suspension of the bacteria was prepared in 2 mL of 0.85% saline to give an absorbance at a wavelength of 490 nm of 0.6 equivalent to a bacterial count of 1.0 × 10^8 ^colony forming units (cfu) per mL and the catheter was filled with this suspension. The remaining three animals were not inoculated. Post-operative monitoring and care was similar to that described for the post-induction recovery period except that no antibiotics were given. The animals were checked daily and data on temperature, heart rate, food and water intake, and incision site were obtained. The animals were weighed weekly, and closely monitored for clinical signs of infection for the four weeks following the shunt inoculation and implantation procedure. These signs included systemic variables such as sepsis and a persistent fever greater than 39.1°C, neurological variables such as lethargy, coma, seizures, and focal deficits, and local variables such as swelling, redness, soreness, and discharge. If no signs of infection occurred during the 4 weeks of observation, the animals were anaesthetized and a dissection was performed under aseptic conditions.

### Shunt removal and sampling

**Figure 1:**
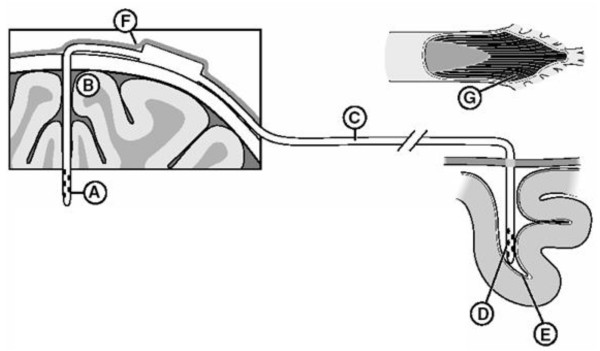
**Diagram of sample sites at necropsy**. A: proximal catheter; B: parenchymal brain tissue adjacent to the catheter; C: distal catheter; D: distal catheter tip in the peritoneal cavity; E: tissue adjacent to distal catheter tip; F: tissue along the catheter track; and G: cerebrospinal fluid from the lumbar subarachnoid space.

The distal end of the VP shunt was carefully exposed by gross dissection and ligated with silk sutures at the ventricular catheter, proximal and distal to the valve, and at the most distal end of the shunt tubing. Biopsies of the brain parenchyma and meninges adjacent to the ventricular catheter track were obtained via a 2 cm bilateral cranial burr hole. A biopsy of the peripheral tissue adjacent to the valve was obtained and the valve removed. The distal catheter tip was cut and removed along with the remaining peritoneal shunt tubing. The three tissue samples (brain parenchyma, tissue adjacent to the valve and peritoneal tissue adjacent to the distal catheter tip), catheter tips (proximal and distal ends), ligated shunt, and the CSF samples (Fig 1) were sent for microbiological analysis (Cleveland Clinic, G.H., M.T.). CSF samples were obtained by lumbar puncture (LP). Each dog was then sacrificed with KCl while under anaesthesia and perfused intravascularly with 4 L of 4% paraformaldehyde fixative (Prills, 95%, Aldrich Chemical Company, Ind., USA) in 0.1 M PBS, via the internal carotid artery. The brain was removed from the skull and stored in cryostorage solution (30% sucrose) until further processing.

### Microbiology

The tissue samples, catheter tips and lumbar CSF samples underwent routine microbiological processing. The ligated shunt tubing was sonicated for one minute, and fluid withdrawn from the lumen for culture. After incubation overnight in CO_2 _at 35°C, bacterial growth and identification for all samples were determined. The isolates were then sent to the University of Nottingham (R.B.) for further examination. After confirmation of identity using basic tests, each isolate was examined by APIStaph (bioMérieux, Marcy L'Etoile, France), numerical antibiogram [18] and pulse field gel electrophoresis (PFGE) (ChefII -DRII, Bio-Rad Laboratories Ltd, Hemel Hempstead, UK).

### Pathology

Each brain and its corresponding meninges was examined for gross signs of encephalitis, meningitis, and ventriculitis (GP). Tissue from the right cerebral hemisphere, which included the ventricular catheter track from each animal was embedded in paraffin wax, sectioned at 8 μm, and stained with haematoxylin and eosin, and the Brown-Brenn tissue Gram stain.

## Results

### Induction of hydrocephalus

All seven animals that underwent the induction of chronic hydrocephalus procedure recovered from surgery. One animal developed symptoms of acute hydrocephalus and required two days of observation in the Critical Care Unit and treatment of raised ICP before resolution. In the remaining animals, vital signs monitored (weight, temperature, heart rate, and food and water intake) during the three to four-week period following the induction surgery remained in the normal range. At the time of sacrifice, Evans ratio of all animals was >0.3 confirming ventriculomegaly. There was no incidence of mortality or morbidity in any animal for either the experimental or control groups. Most hydrocephalic animals exhibited signs of lethargy, motor weakness, anorexia, and ataxia in the first seven days immediately following surgery. Vomiting often accompanied neurological signs (i.e., pupillary dilation and reflex impairment) reflecting increased ICP. These neurological signs were observed to be transient and resolved within the first one to two days. No animal was observed to be febrile. Autopsy performed in each animal revealed no evidence of intracerebral or intraventricular bleeding, which corroborated with MRI data. In addition, the surgical incision site in all animals healed well and did not show any signs of infection.

### Shunting and inoculation

Six of the seven dogs lost weight during the first week following induction of hydrocephalus. The one animal that did not lose weight following surgery and one other animal whose weight returned to normal following recovery from induction, had shown a weight loss two weeks prior to the shunt implantation surgery. Food intake decreased by almost 50% eight days prior to the shunting procedure. In the four-week period following shunting all animals showed a steady weight gain for the remainder of the study. The animals recovered from the shunt-inoculation procedure very quickly. They had normal consumption of food and fluid the first day post-operatively. Gait and vital signs were normal for the four-week period following the inoculation-shunt procedure. None was febrile and there were no signs of infection of the abdominal or head wounds.

### Observations at Dissection

At dissection, the animals were deeply anaesthetized, facilitating close examination of the wounds. In five of the seven animals, upon opening the healed sutured skin on the head, fluid collection was observed. In one animal this fluid was thick and slightly discoloured and followed the shunt catheter distally to the valve (neck region). On removal of the ventricular catheters, a plug appeared to block the end of the catheter in five animals. In addition, there was protein matter (non-brain) infiltrating some of the valve systems. No visual signs of infection were observed at the abdominal insertion incision. At autopsy no peritoneal cysts or other abnormalities were observed.

### Microbiology Results

**Figure 2:**
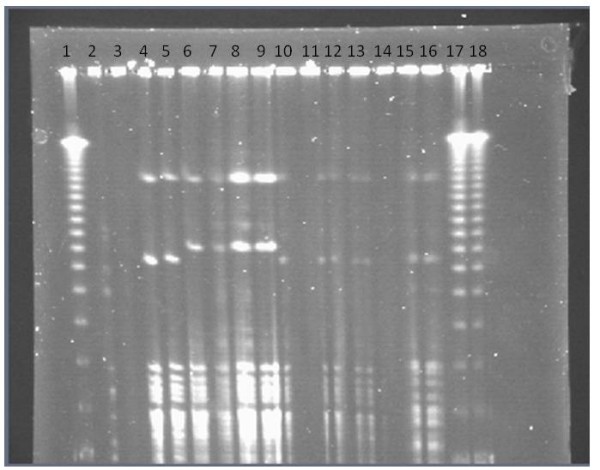
**Results of Pulsed field gel electrophoresis (PFGE)**. Lanes 1(left), 17 and 18 (right) are DNA standards. All other lanes are in duplicate (lane 14 is uninoculated). Lanes 2 & 3 are *Staphylococcus epidermidis *F22, the inoculum bacterium. Lanes 4 & 5 are tissue isolates from Dog 2. Lanes 6 & 7 are isolates from Dog 1 inside tubing, and 8 & 9 from Dog 1 outside tubing. Lanes 10 & 11 are isolates from Dog 3, lanes 12 & 13 from Dog 6 inside tubing. Lanes 15 & 16 are from Dog 7 inside tubing. For the *Staphylococcus intermedius *isolates, two different band patterns can be seen, designated A (Lanes 4, 5, 10, 11, 12, 13, 15 & 16) and B (Lanes 6, 7, 8, & 9).

These are summarized in Table 1. The LP CSF samples (G) showed only a few leukocytes and all were culture – negative. The four animals inoculated with *S. epidermidis *grew this organism from most of the collected samples (dogs 1, 3, 4, 5,). In two of these four "S. epi" animals, a second bacterium was also cultured. This was identified (APIStaph) as *Staphylococcus intermedius*. *S. intermedius *was grown in broth culture only from the brain tissue and proximal catheter samples of one animal and from the peritoneal tissue sample from the second animal. In the remaining three uninoculated control animals (dogs 2, 6, 7), all samples were positive for *S. intermedius*. The *S. intermedius *isolates were compared by APIStaph, numerical antibiogram and PFGE (Fig 2). The results of these were collated and are shown in Table 2. The PFGE, biochemical and antibiogram profiles for F 1568 and F1569 (dog 1, inside and outside the tubing) were identical; F 1567, F 1570, F1571 and F 1572 showed slight differences in one or other profiles. Isolates from dogs 2, 3, 6 and 7 were therefore similar to each other but differed from those from dog 1.

**Table 1:** Culture results from various sites at necropsy.

**Animal**	**A**	**B**	**C**	**D**	**E**	**F**	**G**	**Gross signs**
**1**	Si	Si	Se	Se	NG	NG	NG	+
2	Si	Si	Si	Si	Si	Si	NG	+
**3**	Se	Se	Se	Se	Si	NG	ND	-
**4**	Se	Se	Se	Se	Se	NG	ND	+
**5**	Se	Se	Se	Se	Se	Se	NG	+
6	Si	Si	Si	Si	Si	Si	NG	+
7	Si	Si	Si	Si	Si	NG	NG	-

Sampling sites A-G are shown in Figure 1. Si: *Staphylococcus intermedius*; Se: *Staphylococcus epidermidis *(the inoculated strain). NG: no growth; ND: not done. Gross signs of infection were noted as present (+) or absent (-) at necropsy. The shunts of dogs 1, 3, 4 & 5 (bold type) were inoculated with *S epidermidis*, and dogs 2,6 & 7 remained uninoculated.

**Table 2:** Comparison of the *Staphylococcus intermedius *isolates.

**Isolate number**	**dog**	**identity**	**PFGE type**	**Biochem Profile**	**Antibiogram**
F1567	2	S. intermedius	A	6716113	61000
F1568	1a	S. intermedius	B	6716153	61000
F1569	1b	S. intermedius	B	6716153	61000
F1570	3	S. intermedius	A	6716153	61000
F1571	6	S. intermedius	A	6716153	61200
F1572	7	S. intermedius	A	6716153	61200

Isolates from dogs 2, 1b and 3 were from shunt – associated tissue. Those from dogs 1a, 6 and 7 were from the inside of the shunt tubing (1a and 1b were the same dog). With reference to Fig 2, the two PFGE patterns were designated A and B to distinguish. It may be seen that the PFGE, biochemical and antibiogram profiles for F 1568 and F1569 (dog 1, different sites) were identical; F 1567, F 1570, F1571 and F 1572 showed slight differences in one or other profiles.

### Pathology Results

Histopathological examination revealed no sign of ventriculitis or inflammation. There was minimal amount of gliosis and scarring in the brain tissue surrounding the catheter track similar to and consistent with previous reports and found after insertion of ventricular catheters in the absence of infection. Also, there was no evidence of bacterial invasion on Gram stain.

## Discussion

One of the more striking findings from this study was the survival and overall health of each animal, with weight gain and no signs of systemic infection after shunting. The wounds healed well and there was no general spread of bacteria within the ventricular system and no classic signs of inflammation in the brain. The features of overt shunt infection include fever, with in some cases inflammation over the catheter track and possible wound breakdown. Distal obstruction of the peritoneal catheter, with consequent signs of raised intracranial pressure, would be expected. None of these was seen, and benign pathophysiology and lack of bacterial spread may be surprising in view of the large inoculation with *S. epidermidis*. It could be explained in part by the termination of the experiment at 4 weeks; these low-grade staphylococcal infections often have a longer latency and are more indolent and difficult to diagnose. They cause little or no direct injury, but if untreated they will eventually usually cause distal catheter obstruction due to adhesions or pseudocyst formation [19,20]. However, there is another possible explanation: *S. epidermidis *infections are a challenge to diagnose and treat, in part because of the ability of most strains to grow inside shunts as biofilms [21,22]. Bacteria in biofilms downregulate their synthesis of target sites for antibiotics, thus becoming insusceptible to antibiotic therapy [23]. The biofilm mode may be another explanation for the finding of uniformly positive catheter lumen cultures but no evidence of bacterial spread, invasion, or inflammation in the periventricular brain and ependyma. The negative lumbar CSF cultures as well as the benign central nervous system pathology may indicate the inability of this bacterium to migrate rapidly from the adherent biofilms into the surrounding tissues, though the lumbar CSF would not be likely to show signs of infection in view of the obstructive hydrocephalus. Indeed, the human clinical features of *S. epidermidis *VP shunt infection, especially at four weeks post surgery, show a similar limited response. While there was no evidence of inflammation using standard histological techniques, other markers of inflammation and infection, such as microglial proliferation and changes in cytokine levels in the brain and/or CSF might have been informative. Also, more pathological abnormalities might become apparent in future studies when the infections are allowed to run for several months before sacrifice. This is suggested by the finding of subgaleal fluid collections in five of the animals on dissection.

Using the model a 100% infection rate (four out of four animals inoculated with *S. epidermidis*) was achieved and verified by gross examination and culture of hardware and tissue. In addition, the important role of skin flora in seeding of a shunt system during insertion was demonstrated fortuitously by the high rate of *S. intermedius *isolation, the three dogs that were not inoculated with *S. epidermidis *all contracting shunt infections due to *S. intermedius*. The evidence of infection decreased with samples taken downstream of the catheter in the peritoneal cavity, and none was found in the lumbar fluid.

In spite of the inoculation of 10^8^cfu/mL bacteria into the ventricular catheter in four animals, in two cases the animals' own *S. intermedius *was also isolated, as well as from the three uninoculated controls *. S. intermedius *comprises 90% of all the staphylococci isolated from canine skin [24]. In two cases where both *S. intermedius *and *S. epidermidis *were found in the same shunt system the *S. intermedius *was found in the more proximal portion, and *S. epidermidis *in the more distal part. Given the flow of CSF through the shunt system this suggests the possibility of bacterial contamination from the proximal end down to the distal end. Comparison of the *S. intermedius *isolates by three methods (Table 2) showed that the two isolates from dog 1 (tissue from shunt track and inside of shunt tubing) were identical but different from the isolates from the other dogs. While the differences were only slight, all the other isolates differed from each other in one characteristic or another, suggesting that in each case the bacteria were derived from the dog's own skin flora rather than from cross – contamination from a single source.

*S. intermedius *has been isolated from a majority of healthy canines [24] although it is rarely isolated from humans. However, transmission to humans has occurred, sometimes resulting in clinical infection [25-27]. *S. intermedius *can be confused with *Staphylococcus aureus*. Both are coagulase positive, and produce a heat – stable nuclease, but *S. intermedius *does not produce Protein A and most isolates are clumping factor negative. Some variation can occur. Our isolates were clumping factor positive but coagulase negative using human plasma, and they produced a heat – stable nuclease. Their identification was kindly confirmed by Professor François Vandenesch of the University of Lyon, France.

This paper characterizes a canine model of chronic adult obstructive hydrocephalus. The animals survived well with no mortality and low morbidity, providing the opportunity for physiological studies, pathophysiological analyses and diagnostic studies.

## Conclusion

In spite of the fortuitous endogenous *S. intermedius *infections, which once appreciated should be avoidable in future studies, we suggest that the model described here, perhaps with extended post – infection monitoring, is particularly suitable for the study of the pathogenesis, prevention and treatment of experimental shunt infections.

## Competing interests

The authors declare that they have no competing interests.

## Authors' contributions

RB contributed to the inception and design of the study, carried out the detailed microbiological investigations, and wrote the manuscript. CB and SMD assisted in the surgery and postoperative management and drafted data. MT and GH carried out the initial laboratory examination of removed shunts and tissues. GP carried out the necropsies and the histopathological investigations. MGL contributed to the inception and design of the study, carried out the hydrocephalus induction, the shunt surgery and the clinical examinations at termination, and oversaw the project in USA. All authors have read and approved the final version of the manuscript.
